# Psychometric Properties of the Dutch Contextual Assessment of Social Skills (CASS): An Independent Observational Outcome Measure of Social Skills in Autistic Adolescents

**DOI:** 10.1007/s10803-023-06156-7

**Published:** 2023-11-11

**Authors:** Sakinah Idris, Femke H. F. ten Hoeve, Allison B. Ratto, Susan W. White, Neeltje van Haren, Kirstin Greaves-Lord

**Affiliations:** 1 Department of Child and Adolescent Psychiatry/Psychology, Erasmus MC-Sophia, Wytemaweg 8, 3015 CN Rotterdam, The Netherlands; 2 Department of Psychiatry, Faculty of Medicine, Universiti Teknologi MARA, Jalan Hospital, 47000 Sungai Buloh, Selangor Malaysia; 3 Division of Pediatric Neuropsychology, Center for Autism Spectrum Disorders, Children’s National Hospital, 15245 Shady Grove Road, Rockville, MD USA; 4 Department of Psychology, The University of Alabama, Tuscaloosa, AL 35487 USA; 5 Department of Psychology, Clinical Psychology and Experimental Psychopathology Unit, University of Groningen, Groningen, The Netherlands; 6 Autism Team Northern-Netherlands, Jonx, Department of (Youth) Mental Health and Autism of Lentis Psychiatric Institute Groningen, Groningen, The Netherlands; 7 Yulius Organization for Mental Health, Burg. De Raadtsingel 93c, 3311 JG Dordrecht, The Netherlands

**Keywords:** Autism spectrum disorder, Contextual assessment of social skills, Behavioral observation, Psychometric properties, Social skills intervention, Social interaction skills

## Abstract

The goal of this study was to translate and adapt the original 9-item of the Contextual Assessment of Social Skills (CASS) to a Dutch version and assess its psychometric qualities. Autistic adolescents aged 12 to 18 years (*n* = 99) took part in a randomized controlled trial. In this study, pre-intervention data were utilized. The original CASS was adapted to ensure cultural relevance and the content validity was assessed. Data was used to assess reliability and structural validity, using confirmatory factor analysis. 4-item were added to the CASS during the adaptation to better align with the objectives of the experimental intervention. The original 9-item had inter-item correlations between .01 and .70. The Cronbach’s alpha for the original 4-item total score was moderate (α = .69), while for a 7-item total score, it was high (α = .86). This 7-item total score had a sufficient model fit (Comparative Fit Index = .90). This total score had a significant correlation with the Assertion subscale of the Social Skills Improvement System-Adolescent (SSIS-A) (*r* = 0.26, *p* < .01), and the Social Responsiveness Scale-2 (SRS-2) total score (*r* = − .21, *p* = *.*04*)* indicating sufficient convergent validity. The CASS total score was not correlated with the Repetitive and Restricted Behavior scale of the SRS-2 (*r* = − .08, *p* = *.*43), indicating sufficient divergent validity. The Dutch CASS can be considered a conceptually sound and reliable observational instrument for assessing social conversational skills in Dutch autistic youth. Further evaluation of its feasibility when implemented in practice, outside of clinical research, is needed.

*Trial registration*: Dutch trail register NTR6255 (NL6117) 08/02/2017 https://www.trialregister.nl/trial/6117

## Introduction

Autistic adolescents (N.B. we use ‘autistic’ based on the research by Kenny et al., 2015) may struggle with social interaction and communication e.g., starting and continuing conversations with their peers (Ke et al., 2017). These difficulties may lead to lower quality friendships, higher feelings of loneliness, and more social anxiety than in their typically developing peers (Bauminger, 2002). As a result, social conversational skills have been a major intervention target amongst autistic adolescents. Although research on the effectiveness of social conversational skills interventions has increased, evidence for the efficacy of these interventions is limited, owing to a lack of psychometrically validated outcome measures that capture clinically meaningful changes in social conversational skills.

Behavioral observations may be one of the most objective outcome measures in general social skills assessment, and has several advantages. Employing trained and masked raters to code the frequency of target behaviors may increase sensitivity to specific changes during the observation (e.g., initiation of conversation, sudden topic changes, and silences) (Cunningham, 2012). Yet another advantage of observational assessments is that it does not rely on introspection or self-report, which permits greater flexibility in use (e.g., with those who have limited verbal ability or who struggle to self-report) (Jibril, 2018).

One published rating system designed to assess social skills in autistic individuals is the Contextual Assessment of Social Skills (CASS; Ratto et al., 2011). The CASS is an observational measure used to assess social interaction skills within the context of a conversation in a short dyadic interaction with an unfamiliar and similarly aged peer, who is a confederate asked to follow specific instructions for social engagement with the participant. The conversations are videotaped. Subsequently, nine items relating to conversational skills are used to code participant behavior in the videotaped conversations (i.e., Asking Questions, Topic Changes, Vocal Expressiveness, Gestures, Positive Affect, Kinesic Arousal, Social Anxiety, Overall Involvement/Interest in the Conversation, and Overall Quality of Rapport). Ratings are performed by trained raters who, depending on study needs, can be masked to (unaware of) certain factors, such as diagnostic status, intervention arm, or assessment timepoint (i.e., pre, post).

The reliability and the validity of the CASS have been examined by the original US developers of this instrument (Ratto et al., 2011). Internal consistency amongst all 9 items was high (standardized alpha = .83) and inter-rater reliability (interclass correlation coefficient) was acceptable with a mean value of 0.68. The 4-item total score (i.e., Asking Questions, Topic Changes, Overall Involvement, and Overall Quality of Rapport) was also analyzed separately and was acceptable (standardized alpha = .75). The CASS total score showed good convergent and divergent validity, since it was significantly associated with verbal IQ (*r* = .32, *p* < .04) and theory of mind (*r* = .47, *p* < .002) but not significantly correlated with performance IQ (*r* = .006, *ns*) (Ratto et al., 2011). Correlations were also conducted with autism severity as measured using the SRS (a parent-rated social behaviour questionnaire) within the autism group only, as these data were not available for the control group (Ratto, 2010)*.* Contrary to expectations, this correlation was not statistically significant (r = − .22, *ns*). In a more recent US study, the CASS 4-item total scores were found to be inversely correlated with the Social Affect subscale of Autism Diagnostic Observation Schedule (ADOS-SA) (*r* = − .44, *p* = .02), suggesting that the CASS is a valid measure of social ability (Simmons et al., 2020). Yet, Simmon et al., (2020) also examined the convergent validity of the CASS domains with Social Responsiveness Scale-Social Communication Index (SRS-SCI), but again, the SRS-SCI was not significantly associated with any of the CASS domains. This lack of correlations might be because the SRS-SCI asks to rate behaviors in the previous 6 months, which may not be directly related to the specific behavior during the direct observation, or due to limited power. Previous studies on the psychometric properties of the CASS were limited by their moderate sample size, and all but one study (Rabin et al., 2018) to date have been conducted with US samples.

Despite the growing use of the CASS as an outcome measure, limited research is available on its psychometric properties. Moreover, no clear consensus recommendations have been made regarding the computation of a total score. For example, Dolan et al. (2016) used only the 7 Likert scale items (excluding the count items: Asking Questions, Topic Changes) to compute a total score, (i.e., Vocal Expressiveness, Gestures, Positive Affect, Kinesic Arousal, Social Anxiety, Overall Involvement, and Overall Quality of Rapport). While the original developers and the Hebrew/Israeli version of the CASS used four items (i.e., Asking Questions, Topic Changes, Overall Involvement, and Overall Quality of Rapport) to compute a total score (Rabin et al., 2018; Ratto et al., 2011; Simmons et al., 2020). It would be useful to reach more consensus internationally on the composition of the total score to improve comparability amongst studies.

The CASS has been used in several studies to evaluate the effects of interventions, including the Program for the Education and Enrichment of Relationship Skills (PEERS®) (Dolan et al., 2016; Rabin et al., 2018; White et al., 2015). Although these studies show interesting results regarding the CASS, enquiry of the psychometric properties of the CASS never is the focus. Although CASS has been used in other cultures, these previous studies did not report in detail on the cultural adaption process. The current study therefore fills this gap in the literature, by not only describing the procedures on how the CASS was linguistically translated, but also describing in detail how it was culturally adapted, to ensure the CASS is relevant in the Dutch culture, as well as to provide an example for other cultures. Moreover, the CASS was aligned with the PEERS intervention program, to make it an even more suitable outcome measure in our own randomized controlled trial (Idris et al, 2022), but these examples of creating aligning items might also inspire future studies. Since the CASS was designed as an outcome measure to assess change in response to social skills interventions in general, but was not specifically tailored to the outcomes of the PEERS® social skills intervention, there is not a one-on-one association between the skills taught in PEERS® and the items rated on the CASS. In fact, some items may show change in the unanticipated direction; for instance, it may be that a client is taught to gradually withdraw from a conversation when the conversational partner does not seem interested, rather than ask more questions or change the topic. As such, the content validity could be tailored better to intervention goals. The addition of new items that are directly related to the skills taught in PEERS® may improve the performance of the CASS as an outcome measure with this specific intervention. To our knowledge, literature on improving the content validity of the CASS is not yet available.

Taken together, research on the psychometric properties of the CASS is scarce. Hence, we aimed to contribute to the psychometric evaluation of the CASS, by investigating the reliability and validity of the Dutch CASS. Additionally, we aimed to tailor the content of the CASS more directly to the skills taught in the PEERS intervention, to improve its sensitivity to change following this specific intervention.

## Methods

### Participants

This study involved 99 autistic adolescents who were recruited for the ACCEPT-study to investigate the effectiveness of the Dutch PEERS® intervention, performing a randomized controlled trial (RCT), while utilizing an active control condition i.e., Regulation, Organization, and Autonomy Didactics Training (ROAD) (Idris et al., 2022). The ROAD intervention provides psychoeducation on different adolescence-related themes such as identity/self-acceptance, autonomy in planning activities/schoolwork, physical appearance and changes, regulating emotions, developing friendships, and solo/partnered sexual activities/boundaries. The inclusion and exclusion criteria were (1) having a diagnosis of ASD conform with DSM-IV or DSM-V; (2) enrolment in secondary education; (3) age between 12 and 18 years old; (4) a total and verbal IQ > 70 (assessed with the WISC-IV or WASI); (5) fluent in the Dutch language (verbally and written); (6) motivation to participate in an intervention and in research; (7) no history of major mental illness (e.g., schizophrenia, bipolar disorder, or other types of psychotic disorders) or any visual, hearing or physical impairments that prohibited participation in the study. For further details, please see van Pelt et al (2020) and Idris et al (2022).

### Target Measure: Contextual Assessment of Social Skills (CASS)

The original version of the CASS evaluates participant social skills under two social conditions: (1) an interested condition in which the confederate showed social interest and engagement; and (2) a bored condition in which the confederate demonstrated boredom and disengagement in the conversation. In both conditions, the adolescents had a conversation for three minutes with an unfamiliar confederate, first in the interested condition and then with another confederate in the bored condition (Ratto et al., 2011). We removed the bored condition in agreement with international colleagues (Corbett et al., 2020; Dolan et al., 2016; Rabin et al., 2018). The bored condition was removed from the CASS procedure, consistent with prior studies utilizing the CASS as an outcome measure for the PEERS® intervention (Rabin et al., 2018; White et al., 2015). The PEERS® intervention teaches adolescents to disengage in a situation where to conversational partner looks ‘bored’/uninterested, therefore it was considered an unsuitable and inappropriate outcome measure in the context of a study on the effectiveness of PEERS®. Moreover, the presentation of a “bored” confederate would potentially bias participant expectations and interaction styles for post-test assessment, thus leading to an invalid observation.

Thirteen typically developing adolescents aged between 13 and 23-years-old (male *n* = 6, female *n* = 7) were recruited from local schools as confederates in this study. They received a 1-day training from a certified CASS assessor and coder (KGL). All confederates underwent this formal training preceding their participation in the RCT, during which they received instructions and practiced their actual behaviours in role plays with the researchers and experienced confederates. In this study, there was no use of an extra assessment tool to measure confederate adherence. Rather, we used the CASS items to also observe the confederates’ behaviors and check if those corresponded to the instructions given. Investigating differences with the confederates’ behaviors was however not the primary objective of the current paper and was therefore not included in the current analyses. Yet, when using the CASS as the primary outcome measure in our RCT study, the variation in confederate behaviors was considered as a covariate, please see Idris et al., (2022). Importantly, to ensure that all confederates remained in line with the instructions in their interaction styles, we sent a reminder about of the instructions using an instant messaging application i.e., WhatsApp before each assessment time-point. In line with the CASS guidelines, all interactions were with a similarly aged and opposite biological sex peer. We maintained this policy, because, traditionally, one of the important social-emotional developmental tasks in adolescence is to learn to engage in interactions with the opposite biological sex. In our sample in this era (2017–2019), several participants did not identify with one particular biological sex, with 2 participants being in transition from one gender to the other (Idris et al, 2022). Therefore, we made sure to include confederates displaying a range of gender expressions (i.e., girly, tomboys, boyish, as well as transwoman). For more details, please see (Idris et al., 2022; van Pelt et al., 2020).

### Behavioral Coding

The CASS recording was used to code the verbal and non-verbal behaviors of both the autistic adolescents and the confederates. Behavioral coding was originally comprised of nine rating items: (1) Asking Questions, (2) Topic Changes, (3) Vocal Expressiveness, (4) Gestures, (5) Positive Affect, (6) Kinesic Arousal, (7) Social Anxiety, (8) Overall Involvement/Interest in the Conversation, and (9) Overall Quality of Rapport. The first two items are rated as frequency counts. The other seven items are rated on a Likert scale of 1 = low, 7 = high. Scores below 6 indicate some level of social skills deficit (Ratto et al., 2011). The original developers of the CASS (Ratto et al., 2011) calculated the total score consisting of four primary items (i.e., Asking Questions, Topic Changes, Overall Involvement, and Overall Quality of Rapport) indicative of the conversational skills to assess social skills of the adolescents. Coding was performed by fifteen undergraduate students. They were established reliability as the CASS coders by coding six training videos (i.e., three original US videos of typically developing young adults, and three new Dutch videos of autistic teenagers). We *compared the scores of each* coder against the scores of the original developer and the translators (*i.e., the coding by A.B. Ratto and the coding by KGL and FtH*). The 15 coders achieved at least 80% overall agreement with the original developer and at least 70% agreement on the new Dutch videos. The coders were masked to the time points of the RCT and to the assigned condition (PEERS versus ROAD) to minimize bias. They each viewed and independently coded the videos.

#### Conversation Rating Scale

Immediately after the CASS, the participants and the confederates were asked to complete a brief questionnaire about how they experienced the conversation [the Conversation Rating Scale (CRS)] (Ratto, 2010). The CRS consists of 5 items rated on a Likert scale (1–7) with a total score range from 7 to 35. Internal consistency of the CRS was high in the pilot study (alpha = .92). Although the CRS has been inconsistently used in subsequent studies, it was utilized in the present study, with newly added items on self-perceived social competence and the conversational partner’s interest, to evaluate participants’ perceptions of the success of the conversation overall and their own role in the interaction.

### Diagnostic Measures

*Diagnostic classification* was determined using the Autism Diagnostic Observation Schedule Second Version (ADOS-2; De Bildt et al., 2009; Lord et al., 2012). Module 3 and 4 were used in the current study, based on the developmental age as well as on the adolescents’ language abilities. The total calibrated severity score (CSS) comprised of two subscales: Social Affect (SA) and Restricted, Repetitive Behavior (RRB) ranging from 1 to 10. The “Autism Spectrum Disorder” classification includes CSS scores in the range from 4 to 10, and the “Non-spectrum” classification includes CSS scores in the range from 1 to 3. If the ADOS-2 had been administered in the past 5 years, those scores were extracted from the patient file with permission from the parents. If a recent ADOS-2 was not available, a trained and licensed clinician administered the ADOS-2. Please note here that the ADOS-2 was assessed to obtain a more objective index of ASD severity, but it was not deemed necessary for ‘confirmation’ of clinical diagnoses.

*Cognitive ability (IQ)* If available, information of the IQ was extracted from the patient file with permission from the parents. If the test was administered more than 5 years earlier, the Wechsler Intelligence Scale for Children (WISC-IV; Wechsler, 2003) or the Wechsler Abbreviated Scales of Intelligence (WASI; Wechsler, 1999) was completed.

#### Parent and Self-report Measures of Social Skills for Convergent and Divergent Validity

*Social Responsiveness Scale-version 2 (SRS-2; *Constantino & Gruber, 2012*)* is a 65-item questionnaire with a 4-point scale ranging from 0 (not true) to 3 (almost always true), so total scores ranging from 0 to 195. It measures the severity of social impairment related to ASD (Constantino & Gruber, 2005) and is completed by parents. The SRS-2 (Constantino & Gruber, 2012) is used for children aged 4–18 years and has an acceptable model fit with the two-factor structure of ASD as defined by DSM-5 (Frazier et al., 2014). It provides information for specific symptom items (i.e., social awareness, social cognition, social communication, social motivation, and autistic mannerisms). The SRS-2 was translated to different languages and the results were promising in terms of its reliability and validity. Consistent with the validation studies in other countries, the Dutch version of the parent report SRS-2 demonstrated high internal consistency (Cronbach’s alpha ranged from .92 to .95, good convergent validity (r = .63 with the ADI-R) and was able to differentiate between children with ASD and from the general population (Roeyers et al., 2011). In our current sample, the SRS-2 had a Cronbach’s alpha of .92.

*Social Skills Improvement System-Rating Scales (SSIS; *Gresham & Elliott, 2008*)* were administered to adolescents to assess social skills at home, and adolescents’ interactions with peers. The SSIS has two scales; Social Skills and Problem Behaviors derived from factor analysis. We only used the Social Skills scale, which consists of 46 items. The scale derived communication, assertion, empathy, engagement, and self-control subscales. It has shown to be sensitive to change in social skills amongst cognitively able autistic adolescents in the PEERS® intervention (Marchica & D’Amico, 2016). Psychometric properties were reported by Gresham and Elliott (2008) for parent questionnaires with coefficient alphas above .77 and test–retest reliability above .73. In our current sample, the SSIS parent had a Cronbach’s alpha of .90, while SSIS-Adolescent had a Cronbach’s alpha of .92.

### Procedures

The RCT participants were recruited from three mental healthcare institutions in the Netherlands that provide specialist outpatient care for autistic individuals. Eligible participants were recruited via three methods; (1) directly referred by psychologists/psychiatrists/pedagogues, (2) other mental health institutions referred participants to either one of the three participating centres, and (3) adolescents/parents applied for participation in the study themselves after reading the information on websites, leaflets/posters or on social media or via referral by their general practitioner. Following referral, adolescents and their parents were contacted by phone to inform them about the study and detailed information was sent to them. The study design is described in more detail in van Pelt et al., 2020. Written informed consent of the adolescents and their parents was obtained for all assessments and for the videotaping of the CASS. The pre-treatment assessments were scheduled 1 week before the commencement of the intervention, while participants were not yet assigned to a condition. Before the CASS started, the adolescent was instructed by a research assistant outside of the assessment room. They were prompted to start and end a natural conversation with the unfamiliar conversational partner (i.e., a trained confederate) to “get to know each other”. The participant entered the room after the instruction, where the confederate was already seated. Then, after 3 min, a research assistant knocked on the door as a sign to end the conversation. After the conversation, the participant left the room. The video recorder was placed 5 feet away from the participant and the confederate. Subsequently, the participants as well as the confederates were asked to complete the brief questionnaire about how they experienced the conversation (i.e., the CRS). The CASS conversations were all videotaped for later behavioral coding.

### Study Design

**Fig. 1:**
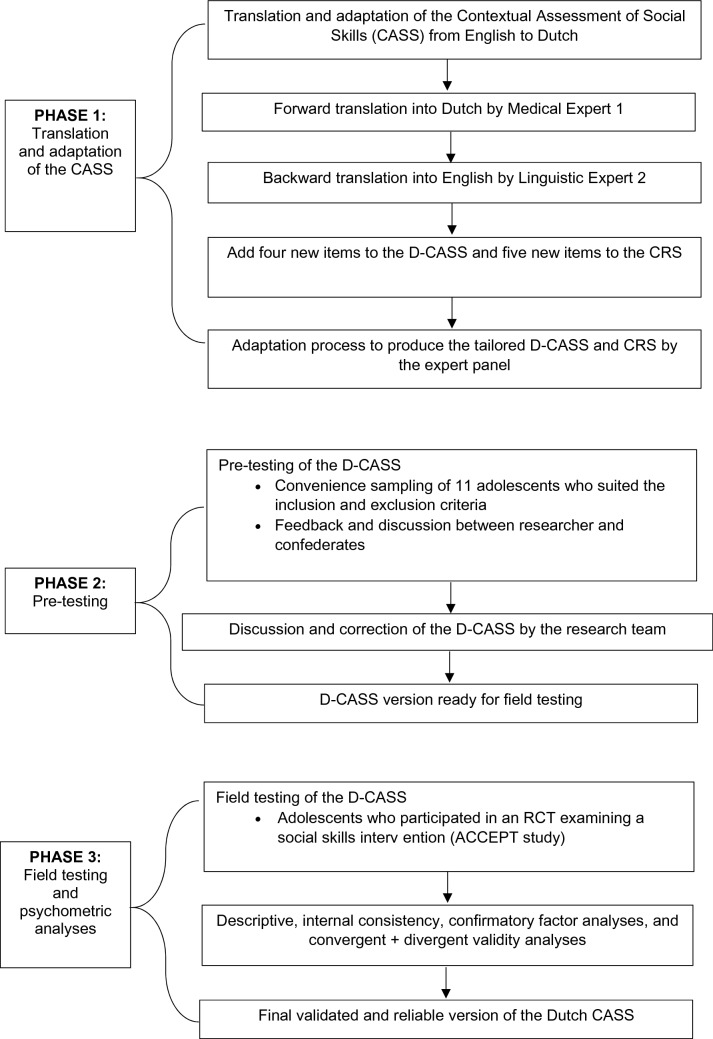
Overview of the study process

This was a cross-sectional assessment validation study. It involved three phases (Hall et al., 2017; Tsang et al., 2017): (i) translation of the CASS and CRS from English to the Dutch language, including cultural adaptation (tailoring content); (ii) pre-testing of the Dutch CASS and CRS; and (iii) psychometric evaluation of the Dutch CASS and CRS which included reliability (inter-item correlations, Cronbach’s alphas and confirmatory factor analyses) and validity (i.e. convergent and divergent). Figure 1 describes the entire study process:

#### Phase 1: Translation and Adaptation of the CASS and CRS

Phase 1 of the study involved first a literal translation process, and a subsequent adaptation by an expert panel. The expert panel consisted of six clinical researchers (KGL, FtH, JvdE, SBI, GJ, and SJ) with expertise in ASD and assessment translation/validation. The panel reviewed the original 9-item of behavioral coding, the CASS manual, and the Conversation Rating Scale (CRS; a self-rated questionnaire on self-perceived competence that comes with the CASS) and the content validity (i.e., alignment with skills usually trained in social skills interventions). The translation of the CASS manual was forwardly translated by FtH and then back-translated by an independent translator (Marianne van der Brugge) according to the guideline recommendations for cross-cultural adaptation and translation studies (Gjersing et al., 2010; Mokkink et al., 2010; Wild et al., 2005). Additionally, four new items were added to the D-CASS (as mentioned above) and five new items to the D-CRS (i.e., two items about self-confidence [before and after the conversation] and three items about the perspective on the conversational partners interest as well as engagement). The D-CRS thus has a total of 10 items, rated on a Likert scale (1–7), with total score ranging 10–70. The internal consistency of D-CRS is alpha = .86.

The forward and back translations were then reviewed, by the other members of the expert panel to address any issues. At the end of phase 1, the Dutch CASS (D-CASS) and Dutch-CRS (D-CRS) version were produced.

#### Phase 2: Pre-testing

The D-CASS and D-CRS were tested on a small group of 11 autistic adolescents from the last two shifts of the pilot, who met the above-mentioned inclusion and exclusion criteria. The adolescents participated in a pilot of two PEERS® groups, which were led and supervised by clinicians trained in the UCLA PEERS® program (GJ & JD). The confederates were trained and coached by KGL. Suggestions for the procedures and coding made by the confederates, clinicians and coders were incorporated. The D-CASS and D-CRS adaptations are described in the results section. After this pilot study, we performed psychometric analyses for the D-CASS and D-CRS on the small set of data from the 11 adolescents. Because of the promising results, we continued with the D-CASS and D-CRS and performed the current larger-scale study.

#### Phase 3: Field Testing and Psychometric Analyses

In phase 3, the D-CASS and D-CRS were field-tested amongst 99 adolescents who participated in the ACCEPT RCT and who fulfilled the same inclusion criteria as the pre-testing. The participants during phase 2 were not re-selected for phase 3 of the study, hence participants for phase 2 and phase 3 were mutually exclusive. Data collected were then subjected to psychometric analyses. First, we evaluated the reliability by computing inter-item correlations and Cronbach’s alpha’s. Then, a confirmatory factor analysis (CFA) was performed using maximum likelihood estimation to investigate the goodness-of-fit between the CASS total score summed of 4-item and 7-item models. Finally, a good model fit was used to examine convergent and divergent validity. A good model fit is indicated by values of 0.90 or higher for the comparative fit index (CFI) and the Tuckerlewis index (TLI) (Bentler, 1990). For the root-mean-square error of approximation (RMSEA), values of 0.05 or lower indicate a close fit, while values less than 0.08 indicate an acceptable fit (Browne & Cudeck, 1993). We used the Social Skills scale of Social Skills Improvement System-Rating Scales (SSIS; Gresham & Elliott, 2008) to examine concurrent validity and RRB subscale of the Social Responsiveness Scale-Version 2 (SRS-2; Constantino & Gruber, 2012) to examine divergent validity.

### Ethical Considerations

The study was approved by the medical ethical commission of the Erasmus Medical Centre, Rotterdam (MEC-NL.57472.078.16) following the Helsinki Declaration of 1975.

### Statistical Analyses

Statistical Program Social Sciences version 27.0 and AMOS version 28.0 were used to analyze data (IBM Corp, 2016). Before conducting the primary analyses, the data were examined for accuracy, missing values, outliers, and multivariate assumptions. Descriptive statistics were used to illustrate the demographic and diagnostic characteristics of the participants and responses to the items in the assessment. Inter-item correlations were calculated for all items. We calculated Cronbach’s alphas (α) to assess the reliability of a 4-item versus 7-item total scale, including the original 9 items. High internal consistency represents Cronbach’s alpha of .70–.80. Cronbach’s alpha between .60 and .70 indicated moderate internal consistency while .5–.60 is considered low internal consistency (Field, 2016; Tavakol & Dennick, 2011).

Subsequently, confirmatory factor analyses (CFA) were performed using maximum likelihood estimation to investigate the goodness-of-fit between the methods to calculate the CASS total score summed of 4-item (Rabin et al., 2018; Ratto et al., 2011) and summed of 7-item (Dolan et al., 2016). Several statistics were used: the Chi-square statistics (χ^2^), the CFI, the TLI, and the RMSEA. Then, the best model was used to calculate the convergent and divergent validity of the CASS. Before the correlations were examined using the Pearson correlation coefficient, the data were checked for any violations of the assumptions of the correlational analyses. No violations were found.

## Results

### The Translation, Adaptation, and Pre-testing

Originally, the CASS consists of nine rating items: (1) Asking Questions, (2) Topic Changes, (3) Vocal Expressiveness, (4) Gestures, (5) Positive Affect, (6) Kinesic Arousal, (7) Social Anxiety, (8) Overall Involvement/Interest in the Conversation, and (9) Overall Quality of Rapport. Besides the above mentioned original nine rating items, the Dutch developers have added four additional items to the D-CASS. The original item 1 was separated between (1a) Initiating and (1b) Follow-up Questions, as these behaviors are specifically instructed within PEERS®. The other three new items are binary coded items (i.e., yes/no), namely (0) Starting the Conversation, (10) Initiating the end of the conversation, (11) Giving a reason to end the conversation. The decision to add the new items was made based on the suggestions from earlier research and international experts to fit more closely to the PEERS® learning objectives. For example, initiating a conversation might depict some form of self-confidence. Floyd and Burgoon (1999) found that participants who initiate the conversation showed the most nonverbal liking behavior responses to the confederate. Obviously, the behaviors of participants at the beginning of a conversation may not tell the whole story. Therefore, we also include the items associated with ending the conversation, especially since these skills are also taught within PEERS.

First, an independent native Dutch language expert carried out the forward translation of the CASS rating manual. Then, the backward translation into the English language was carried out by another independent translator. Besides the translation of the CASS rating manual, the Dutch assessment procedure has also been adjusted. Originally, the procedure was introduced as a role-play. Based on the input of trained confederates from Phase 2, it was decided that introducing it as a natural, ‘getting-to-know-each other’ conversation is less stressful and is feeling more naturally. Second, the two original conditions of the CASS, the interested and the bored condition have been replaced by one interested condition in the Dutch version, consistent with prior trials using the CASS as an outcome for PEERS. Third, the behavioral coding forms of the Dutch CASS provide some space to write down whether a participant asked questions or made statements which were too personal or offensive (verbal content alert). Besides, the forms also provide space to write down some nonverbal inappropriateness (non-verbal behavioral alert) like getting too close, too amicable touches or inappropriate nonverbal behavior for the situation like staring. Fourth, the confederate initiated the conversation after 5-s instead of 10-s in the original CASS. Fifth, after a knock on the door, the adolescents with ASD got the opportunity to finalize the conversation. Finally, the behaviors of the confederates were also rated within the Dutch version. All these elements have been added to the Dutch rating manual by FtH.

The study also extended and adapted the CRS to create the D-CRS. Two items about self-confidence and three items about the perspective taking of the conversational partner on the conversation were added, to align more closely to the learning goals of the PEERS® program. The reason for adding two items on self-confidence is to (a) first measure their feeling/idea of trust in their own ability, probably based on their current self-esteem (potentially increased/decreased during intervention) with the item assessed before the actual conversation, and then (b) to assess their own (potentially more objective) judgement of their actual performance, with the item assessed after the conversation.

### Field Testing and Psychometric Analysis

The percentage of missing values was minimal (< 5%). Because the number of missing values was small, pairwise exclusion of missing data was used to deal with the missing values. The normality of distributions was inspected using histograms and normal q–q plots. Because the distributions were normal, variable transformations were considered unnecessary. The Mahalanobis distance was used to identify multivariate outliers using p < .001 and no outliers were identified.

Out of 106 autistic adolescents who participated in the RCT, and who completed the initial D-CRS items before the start of the actual conversation, seven adolescents were excluded from the analyses on the D-CASS observational items, because there was no useful D-CASS video recording. Of these seven individuals, two adolescents—after filling out the D-CRS self-confidence item, subsequently both refused to perform the actual conversation, because they had realized they were too anxious/unconfident. For the other five adolescents, we did not have a suitable D-CASS video recording due to technical problems (*n* = 2) or because of the confederate not turning up in time *(n* = 3). Therefore, in total, reliable data on the D-CASS observational items were available for *n* = 99. Table 1 shows the descriptive data from the sample (n = 99) whose conversations were observed and coded.

**Table 1:** Means and standard deviations for demographic information (*n* = 99)

	*N*	%	*M* (*SD*)
Age			14.66 (1.53)
Gender			
Male	69	69.7	
Female	30	30.3	
Birth country			
Netherlands	93	93.9	
Belgium	2	2.0	
China	1	1.0	
Others	3	3.0	
Relationship with parents			
Biological	92	92.9	
Foster	3	3.0	
Adoption	3	3.0	
Grandparent	1	1.0	
Special education			
Yes (No)	65 (27)	65.7 (27.3)	
ADOS-2 CSS	74		5.53 (2.46)
Total IQ	80		103.45 (17.27)
Performance IQ	81		100.43 (16.14)
Verbal IQ	81		105.15 (13.09)
SRS-SCI	98		71.80 (20.17)
SRS-RRB	98		14.73 (6.00)
SSIS-A	86		84.65 (18.23)

	N	Range	M (SD)
CRS total adolescents CRS total confederate	98* 99	14–35 12–35	26.59 (4.62) 25.00 (5.90)

*ADOS-2* autism diagnostic observation schedule second version, *IQ* intelligence quotient, *CSS* calibrated severity, *SSIS-A* social skills improvement system-adolescents, *SRS-SCI* Social Responsiveness Scale-social communication and interaction, *SRS-RRB* restricted interests and repetitive behavior, *CRS* Conversation Rating Scale

*1 adolescent had missing data on the CRS

### Reliability

#### Inter-Item Correlations

The inter item correlations of the eleven items of the D-CASS were inspected to see which items correlated significantly with each other. Table 2 shows that the new additional items (i.e., Items 0, 1a, 1b, 10, and 11) did not strongly correlate with the other original items. Items 1 and 2 also did not strongly correlate with the other items. Since removal of these items resulted in a higher Cronbach’s alpha, we decided to work with a total score of 7 items, in line with Dolan and colleagues (2016).

**Table 2:** Inter item correlations of the CASS rating domains

	0	1	1a	1b	2	3	4	5	6	7	8	9	10
0 Starting conversation													
1 Asking questions	.19												
1a Initiating questions	.37**	.63**											
1b Follow-up questions	.00	.87**	.17										
2 Topic changes	.30**	.48**	.78**	.11									
3 Vocal expressiveness	.31**	.34**	.28**	.26**	.14								
4 Gestures	.25*	− .03	.03	− .06	− .02	.44**							
5 Positive affect	.34**	.23*	.14	.21*	.06	.71**	.51**						
6 Kinesic arousal	.25*	.33**	.22*	.28**	.29**	.38**	.21*	.33**					
7 Social anxiety	.27**	.29**	.24*	.21*	.24*	.48**	.29**	.43**	.58**				
8 Overall involvement	.36**	.39**	.26**	.33**	.16	.66**	.41**	.72**	.32**	.59**			
9 Overall quality of rapport	.22*	.27**	.20*	.22*	.07	.56**	.38**	.65**	.30**	.60**	.75**		
10 Initiating end of conversation	− .00	.17	.10	.16	.21*	.04	− .02	.09	.17	.07	.20	.20	
11 Giving reason to end conversation	.02	.09	.07	.07	.08	.08	.01	.14	.18	.14	.17	.19	.35**

*p < .05, **p < .01

#### Internal Consistency

In our sample, a high Cronbach’s alpha (α) value was obtained for the D-CASS total score consisting of the sum of the *seven* original rating items of the D-CASS (Cronbach’s alpha = .86), as used in the study of Dolan (Dolan et al., 2016). Meanwhile, Cronbach’s alpha for the *four* items as used by the original developer Ratto et al. (2011) and Rabin et al. (2018) had a moderate internal consistency (Cronbach’s alpha = .69).The internal consistency for D-CRS is high (Cronbach’s alpha = .83).

#### Confirmatory Factor Analysis

To confirm the factorial validity of the D-CASS total score based on the previous studies (Dolan et al., 2016; Rabin et al., 2018; Ratto et al., 2011), we compared the 4-item and 7-item models on their goodness-of-fit using the data from the present study (see Table 3).

**Table 3:** Results of the comparison of different factorial models for the Dutch CASS total score

Model	No. of items	*χ*^2^(*df*)	CFI	TLI	RMSEA	Coefficient
Ratto et al., 2011 & Rabin et al., 2018	4	23.84(2)	.81	.05	.32	.16–1.0
Dolan et al., 2016	7	49.05(14)	.90	.79	.15	.44–.88

*SRMR* standardised root-mean-square residuals, *CFI* Comparative Fit index, *TLI* Tuckerlewis Index, *RMSEA* root-mean-square error of approximation

After estimating the models, goodness-of-fit statistics were obtained. The 7-item model showed a better fit, with CFI and TLI above .90. These findings provided further support for use of the 7-item total score rather than the 4-item total score.

### Validity

#### Convergent and Divergent Validity

Based on results of the previous analyses (i.e., inter-item correlation, internal consistency, and CFA) indicating better fit for the 7-item model, this model was used to evaluate the validity of the D-CASS. The correlations between the D-CASS total score with the subscales of the self-report SSIS-Adolescent, SRS-SCI (parent-report), SRS Autistic Mannerism, and the Verbal IQ at baseline are shown in Table 4.

**Table 4:** Correlations between CASS total score, SSIS-adolescent total score, SSIS subscales, SRS-SCI, and SRS-2 RRB

	SSIS-A	Comm	Coop	Ass	Resp	Emp	Eng	Self	SRS-SCI	SRS-RRB	VIQ
Pearson *r* Sig. (2-tailed)	.12 .24	.14 .18	− .02 .82	.26 .01*	.12 .24	.10 .34	.16 .12	.05 .65	− .21 .04*	− .08 .43	.23 .04*

*SSIS-A* social skills improvement system-adolescents, *SRS-SCI* Social Responsiveness Scale-social communication and interaction, *SRS-RRB* restricted interests and repetitive behavior, *Comm* communication, *Coop* cooperation, *Ass* assertion, *Resp* responsibility, *Emp* empathy, *Eng* engagement, *Self* self-control, *VIQ* verbal IQ

*p ≤ .05

Regarding the convergent validity, the 7-item D-CASS total score was significantly correlated with the SSIS sub-subscale Assertion (*r* = .26, *p* = .01) and with the SRS-SCI (*r* = − .21, *p* = .04). Regarding the divergent validity, the D-CASS total score was not significantly correlated with the RRB subscale (*r* = − .08, *p* = .43), indicating that the D-CASS is a measure of social skills and not of other autistic symptoms. The D-CASS Total and Verbal IQ are significantly correlated (*r* = .23, *p* = .04).

## Discussion

The purpose of this study was to adapt the CASS to the Dutch population and to evaluate the psychometric properties of the D-CASS using confirmatory factor analyses (CFA). The translator and the expert panel all agreed on the translation during the translation process. Some behavioral coding modifications were done to create the D-CASS. Four new binary items; Initiating and Follow-up Questions, Starting the Conversation, Initiating the End of the Conversation, and Giving Reason to End the Conversation were added to better align with the social skills learning objectives of PEERS.

The first two count items of the original CASS (i.e., Item 1: Asking Questions and Item 2: Topic Changes) were not well correlated with the other items during the analyses. Aside from that, the frequency count items may be incompatible with the specific skills and social customs taught in PEERS® (e.g., the rule “don’t be an interviewer”), that conflict with counting the number of questions asked as an index of social skills, since asking too many questions might be considered too interruptive/dominant (Dolan et al., 2016; Laugeson & Frankel, 2010). Therefore, in this study, these two frequency items were not integrated into the CASS total score. The four additional new items were also not included in the D-CASS total score, because these items are on a binary scale, whereas the other seven original items are a 7-point Likert scale. A combination of these scales may bring to a low/high variability level as well as floor and ceiling challenges (Grassi et al., 2007). In a paper about the outcomes of the Dutch version of PEERS®, we did report on these items (Idris et al, 2022). In the near future, in consultation and collaboration with all international researchers who use the CASS, we will discuss the (potential) value of the additional new items that were introduced in the Dutch CASS for broader purposes, on which we intend to co-create a follow-up paper (i.e., on the shared creation of the international CASS-2).

Two distinct item sets have been used to calculate the CASS total score: the original 4-item set (Ratto et al., 2011) and a 7-item set (Dolin et al., 2016). The two models were compared in terms of psychometric properties. The 7-item model produced more robust fit indices (e.g., CFI), relative to the 4-item model that showed relatively low CFI, TLI and the RMSEA (Table 2).

The Dutch CASS total score was modestly correlated with the SSIS Assertion subscale, assessing a person’s capacity to effectively express feelings, wants, and desires, which is crucial during a conversation. This suggests that the D-CASS has sufficient convergent validity and could be a useful treatment outcome measure for adolescents who need to enhance their social skills. Divergent validity was also evaluated. In line with Ratto et al. (2011), the CASS total score was not significantly correlated with the SRS-autism mannerism subscale, indicating sufficient divergent validity.

Apart from demonstrating that the Dutch CASS is a reliable and valid observational measurement, the current study has other important strengths. First, the current study is the first to go through a translation and validation procedure based on established guidelines (Hall et al., 2017; Tsang et al., 2017) in the Dutch population with a large sample (*n* = 99). Second, it should be noted that the sample employed in this study, can be regarded a good representation of the Dutch autistic population in terms of generalizability (94% are Dutch, from ranging areas of the country). The current study’s sample was heterogenous and included both boys and girls. Boys and girls with ASD are represented in the Dutch society in a 4:1 ratio (Nederlands Jeugdinstituut, 2015) and the sample was drawn from various mental health institutions around the Netherlands.

Aside from the benefits mentioned above, the current study had certain drawbacks. First, there is always a small possibility that there might be slight rater bias in the coding of CASS videos. Even though the time point of the conversation and the intervention condition of the autistic adolescents were meticulously kept hidden from the raters, the raters sometimes were smart members of the RCT research team, therefore, sometimes they may have used specific information about the project organization (i.e. specific location of assessments/trainings, starting date of the project/season) to brightly reason about group-membership (i.e. condition) and/or timepoint (pre, post or follow-up) during their video coding. In this study, we only used the pre-assessment videos; nonetheless, some raters may have subconsciously been slightly biased to give these pre-assessment videos lower scores. Second, the logistics and implementation of the CASS are both complex and time-consuming. For example, finding suitable confederates and training the coders to reliably score the CASS takes considerable time and effort. Therefore, at some rare occasions, there was a larger age difference between conversational partners than would have been ideal. Furthermore, participating in the CASS can be anxiety-provoking for certain participants, as illustrated by the two participants who along the way declined to participate. They were asked to start a conversation with a stranger while being recorded. Some of the participants became overwhelmed (had a black out) and some might have become more cautious (acting very shy or nicer), as reported in the CRS. Therefore, these issues should be taken into consideration when implementing the D-CASS.

Our findings also provide points for consideration in future treatment research that will use the CASS or similar observational measures. Confederates were trained during the trial to ensure the uniformity in procedures and social communicative behavior of the confederates during the CASS. This training is essential, as it may help ensure reliability across participants and time points. Here we provide two suggestions to improve consistency during the CASS procedures and to ensure that the conversations are truly social and reciprocal in nature: First, we modified the instruction given at the beginning of the assessment outside the room rather than inside the room (e.g., “You will have 3-min to talk and get to know each other. After 3-min, I will knock on the door and both of you need to finalize the conversation”). In comparison the previous versions, in this way, there is no test leader present in the room. Second, we changed the procedure for finalizing the conversation. Rather than coming into the room and interrupting the ongoing conversation, after 3 min the test leader knocked on the door and the autistic adolescents got the opportunity to themselves finalize the conversation. The confederates were asked to leave the finishing of the conversation to the participants, to allow the participants to demonstrate their conversation finishing skills (i.e., in line with the social etiquette as taught during PEERS). If the conversation paused, confederates were told to wait 5-s before reinitiating the conversation.

This study may contribute to the critical need for an observational measurement for assessing the efficacy of social skills interventions. Therefore, the current study was a preliminary step in describing the Dutch CASS and providing a foundation for future, larger international studies. In the future, social skills interventions may be evaluated using observations rather than questionnaires. The CASS videos could also be incorporated into social skills interventions as a video feedback instrument for autisticindividuals, assisting them in reflecting on their social skills and identifying concrete goals to work on during the intervention. Obviously, such a clinical implementation would be time-consuming and thus expensive, but it could improve the efficacy of the intervention and thus be worth considering for future innovations.

## Conclusion

This study used pre-assessment RCT data to investigate the reliability and validity of the Dutch CASS. Results suggested that a total score of 7-item had the best Cronbach’s alpha and a sufficient CFI. Other researchers should however conduct their own reliability and factor analyses to assess which total score is most appropriate in their dataset. Consensus on how to consistently use and present results of the CASS will be important to establish if this instrument is to be used to compare results amongst studies.

The D-CASS has the potential to be a suitable treatment outcome measure for evaluating the outcomes of social skills interventions for two reasons: (1) it reflects the most common social obstacles struggled with by autistic adolescents; (2) it is a direct assessment of an individual’s social interaction with similarly aged peers, which is a difficult task for individuals with ASD (White et al., 2015). Findings from this research and other research (Corbett et al., 2020; Dolan et al., 2016; Rabin et al., 2018; Simmons et al., 2020) using CASS as the outcome indicate that the CASS is a feasible alternative in research settings. As a social interaction skills measure, the CASS allows autistic adolescents to practice in an engaging, semi-structured, and supportive environment. It is likely that this planned reciprocal social interaction activity will also help set the stage for interactions with peers in other social settings, such as at home, at the playground, and in community environments, as usually reported on by the parents (e.g. using the SRS-SCI).

Finally, the use of a peer-mediated approach in interventions for autistic children and adolescents showed positive outcomes (Barry et al., 2003; Kamps et al., 1992; Kasari et al., 2012; Lang et al., 2011; Odom & Strain, 1984). Similarly in the CASS, the confederates were trained and supervised peers, who delivered learning opportunities based on the intervention protocol with a high degree of reliability and competence. Utilizing the CASS as a clinical tool besides the purpose of a research outcome measure is therefore estimated to be of high value.

## Data Availability

The datasets generated and/or analyzed during the current study are not publicly available but, are available from the corresponding author on reasonable request after the results are published. The policy of Erasmus Medical Centre is that sharing data with parties outside the organization requires a personalized data transfer agreement between Erasmus Medical Centre and these parties. This requirement prohibits publishing data in freely accessible depositories. Only authors have access to the data.
